# Documentation and diagnosis of delirium in Parkinson's disease

**DOI:** 10.1111/acps.13470

**Published:** 2022-07-19

**Authors:** Rachel J. Cullinan, Sarah J. Richardson, Alison J. Yarnall, David J. Burn, Louise M. Allan, Rachael A. Lawson

**Affiliations:** ^1^ CNTW NHS Trust Newcastle upon Tyne UK; ^2^ Translational and Clinical Research Institute Newcastle University Newcastle upon Tyne UK; ^3^ Faculty of Medical Sciences, NIHR Newcastle Biomedical Research Centre Newcastle University Newcastle UK; ^4^ Newcastle upon Tyne NHS Foundation Trust Newcastle upon Tyne UK; ^5^ Faculty of Medical Sciences, Population Health Sciences Institute Newcastle University Newcastle upon Tyne UK; ^6^ Centre for Research in Ageing and Cognitive Health University of Exeter Exeter UK

**Keywords:** confusion, delirium, diagnosis, Parkinson's disease

## Abstract

**Objective:**

To assess the accuracy of documentation of the symptoms and diagnosis of delirium in medical notes of inpatients with Parkinson's disease (PD).

**Methods:**

The DETERMINE‐PD pilot study assessed PD inpatients over 4‐months. Delirium prevalence was classified prospectively using a standardized assessment at a single visit on the basis of Diagnostic and Statistical Manual of Mental Disorders 5th Edition (DSM‐5) criteria. Incident delirium was diagnosed retrospectively using detailed clinical vignettes and validated consensus method. Inpatient medical notes and discharge summaries of those with delirium were reviewed for documentation of symptoms, diagnosis and follow‐up.

**Results:**

Forty‐four PD patients consented to take part in the study, accounting for 53 admissions. We identified 30 cases (56.6%) of delirium during the participants' stay in hospital. Of those with delirium identified by the research team, delirium symptoms were documented in the clinical notes of 72.3%; 37.9% had a delirium diagnosis documented. Older patients were more likely to have delirium (*p* = 0.027) and have this diagnosis documented (*p* = 0.034). Time from documentation of symptoms to diagnosis ranged from <24 h to 7 days (mean 1.6 ± 4.4 days). Hypoactive delirium was significantly less likely to have been identified and formally diagnosed (63% of not documented were hypoactive vs. 37% hyperactive, mixed or unclear, *p* = 0.016). Only 11.5% of discharge summaries included diagnosis of delirium.

**Conclusion:**

Delirium in PD is common. Documentation of symptoms of delirium was common; however, fails to lead to a documentation of diagnosis in over half of admissions with delirium and was even less commonly communicated in the Primary Care discharge summaries. This highlights the need for increased education about delirium symptomatology and diagnosis in PD.

**Significant Outcomes**

- Delirium documentation in Parkinson's disease is poor.
- Hypoactive delirium is the most commonly missed subtype of delirium.
- Communication by discharge summary of delirium to primary care is extremely poor and increases the difficulty with identifying patients at risk of further delirium and/or dementia.

**Limitations**

- Our study cohort was small and therefore power of the results is limited.
- We have not, within this study, matched with controls of delirium diagnosis in patients without Parkinson's disease.

## 1 INTRODUCTION

Parkinson's disease (PD) is a common neurological condition that is defined by the presence of motor symptoms—bradykinesia plus at least one of either tremor, rigidity, or postural instability.^1^ In the recent years, there has been increasing focus on the non‐motor features, including depression, anxiety, cognitive impairment, delusions, and hallucinations. Whilst much of the literature focuses on PD psychosis, there has been a rise in interest into the prevalence, characteristics and treatment for delirium in PD.^2^, ^3^

Delirium is a neuropsychiatric syndrome that is defined by the Diagnostic and Statistical Manual of Mental Disorders 5th Edition (DSM‐5) as a disturbance of attention, awareness and cognition which develops over a short period of time as a direct consequence of another medical condition, medication, or substance intoxication or withdrawal.^4^ Delirium is often categorized by motor subtype into either hypoactive (reduction in psychomotor activity), hyperactive (increase in psychomotor activity) or mixed (variable levels of psychomotor activity such that neither increased or decreased is predominant).^5^ Delirium severity can be measured using tools such as the Memorial Delirium Assessment Scale^6^ and whilst hyperactive delirium tends to score higher, hypoactive delirium is associated with greater morbidity and mortality.^7^

Delirium is common, occurring in 29%–64% of patients on general medical and old age medicine wards.^8^ However, the diagnosis remains frequently missed, due to under diagnosis and under documentation.^9^, ^10^ Delirium has a significant impact on morbidity and mortality, with increased risk of falls, institutionalization, dementia and mortality.^11^, ^12^, ^13^, ^14^ Identification of delirium is essential to allow targeted investigation for underlying causes, treatment of these and management of the delirium itself, with delays in treatment of delirium associated with increased mortality rates.^14^, ^15^ It is also important to remember that delirium is not only treatable but preventable, but also so early identification of risk factors and early symptoms could have significant impact on reducing morbidity and mortality.^8^

Parkinson's disease is recognized as a risk factor for developing delirium but the prevalence rates have been found to vary greatly, with a range of 0.3%–60% across studies within different settings.^2^ Overlapping symptoms between PD and delirium create diagnostic difficulties. Symptoms common in both conditions include disturbance of attention and awareness, cognitive disturbances, fluctuations in cognition, visual hallucinations, sleep disturbance, daytime somnolence, falls, mood disturbances, and delusions.^2^ The diagnostic challenge of delirium in PD highlights the need for further research into its presentation and characteristics, to better understand how we can identify delirium in these patients. The “Identifying delirium in people with Parkinson's disease (DETERMINE‐PD)” pilot study found that delirium is common in PD inpatients at admission and the incidence increased during participants' hospital stay.^16^ We used these data to assess the accuracy of delirium diagnosis and symptom documentation in medical notes of inpatients with PD compared with those documented from systematic assessment by a research team. By understanding more about how accurately delirium in PD is currently documented by health‐care professionals this can help guide production and targeting of educational tools and materials.

### 1.1 Aims of the study

To assess the documentation of symptoms and diagnosis of delirium in patients with PD during acute hospital admissions. We hypothesized the documentation of the diagnosis of delirium in medical notes of PD participants would be low, a lack of symptom documentation would be associated with lack of recorded diagnosis and hypoactive delirium would be most frequently missed.

## 2 METHODS

### 2.1 Participants

All patients with PD attending Newcastle Upon Tyne Hospitals movement disorders clinic were invited to be included on an electronic Recurring Admissions Patient Alert System (RAPA). All patients who accepted and were admitted between 26 March and 25 July 2018 were then approached in hospital and invited to take part in the study.^16^ Inclusion criteria comprised a diagnosis of PD according to the U.K. Brain Bank Criteria^1^ made by a movement disorder specialist and a hospital admission during the recruitment period. Patients were excluded if they did not have a diagnosis of idiopathic PD; were near death; lacked capacity to give informed consent and no appropriate consultee was available; or had insufficient English to complete the assessments. Patients who were assessed as having capacity according to the Mental Capacity Act 2005^17^ completed written informed consent forms and those assessed as lacking capacity had a personal consultee identified to complete a consultee declaration form. The study was approved by the Yorkshire & Humber‐Bradford and Leeds Research Ethics Committee.

### 2.2 Measures and assessments

A single study assessment visit with participants was completed in hospital as soon as practicable following admission. A collateral history was gained from participants' relative or carers. Demographic information was collected and Clinical Frailty Scale (CFS) completed.^18^ Delirium severity was measured by the Memorial Delirium Assessment Scale (MDAS)^6^ and levels of arousal were assessed using the Observational Scale of Level of Arousal (OSLA)^19^ and Modified Richmond Agitation and Sedation Score (m‐RASS).^20^ PD motor severity was assessed using the Movement Disorder Society Unified Parkinson's Disease Rating Scale Part III (MDS‐UPDRS III).^21^ Levodopa Equivalent Daily Doses (LEDD) were calculated using conversion formulae from Tomlinson et al.^22^

Prevalent delirium was diagnosed prospectively during the single research assessment, whilst in hospital using a structured, standardized assessment of PD participants and collateral history from relatives/carers based on the DSM‐5 criteria.^13^ Incident delirium was diagnosed using detailed clinical vignettes compiled from participants' medical notes, and a validated consensus method.^23^ All diagnoses of delirium were subcategorized into hypoactive, hyperactive, mixed, or unclear.

### 2.3 Medical notes review

We reviewed the medical and nursing notes of those identified as having probable or likely delirium during their hospital admission. The presence of ”delirium” by health‐care professionals was recorded as a diagnosis of delirium having been made. Terms referring to ”confusion,” ”disorientation,” ”cognitive impairment,” and ”hallucinations” were included as delirium symptoms. The date, and type, of the first symptom by any health‐care professional and date of ”delirium” written in the medical notes by a doctor was used to calculate the time in days from symptom documentation to documentation of a formal diagnosis, where a formal diagnosis was recorded. We recorded any involvement with the liaison psychiatry team during admission and the reason for their involvement. Discharge summaries were reviewed for any diagnosis of delirium or plan for any relevant follow‐up for further cognitive assessment or review.

### 2.4 Statistical analysis

Results were collated in the SPSS (Version 25.0; SPSS, Armonk, NY: IBM Corp). Data were examined for normality of distribution with visual histograms and Kolmogorov–Smirnov's test. Comparisons of means between two groups were performed with Mann–Whitney U tests or independent t‐tests, as appropriate. Pearson Chi‐squared tests were used to compare between‐group distributions of proportions. Dates from medical notes were used to calculate times between documentation of symptoms and diagnosis. All statistical tests were two‐sided with statistical significance set at *α* = 0.05.

## 3 RESULTS

**FIGURE 1:**
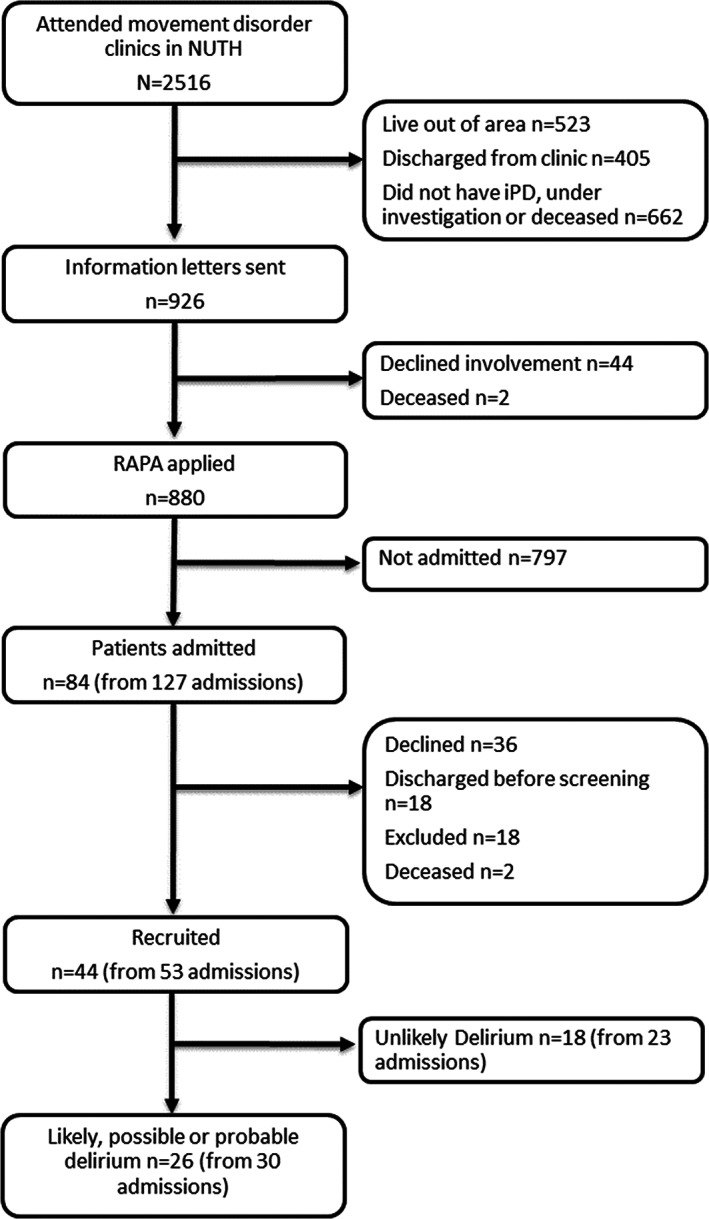
Flow diagram of recruitment. NUTH, Newcastle Upon Tyne Hospitals; iPD, idiopathic Parkinson's disease; RAPA, recurring admissions patient alerts system.

There were 53 admissions from 44 participants enrolled during the DETERMINE‐PD study period (Figure 1). Thirty admissions (55.6%, *n* = 26 participants) were identified as having possible or probable delirium at some stage during their inpatient stay (Table 1). Compared with those without delirium, participants with possible or probable delirium were significantly older (*p* = 0.027), frailer (*p* < 0.001), and had longer hospital stays (*p* = 0.004, Table 1). The mean time between admission and the single study visit was 47.8 ± 32.3 h. Mean length of hospital stay overall was 14.7 ± 15.14 days. Liaison psychiatry referrals were made in only three (10%) delirium admissions; all had a mixed delirium and the reason for liaison referral was for advice and support for low mood or suicidal thoughts.

**TABLE 1:** Comparison of demographics and clinical characteristics of admissions with and without delirium

	Delirium *n* = 30		No delirium *n* = 23				Delirium Diagnosis documented, *n* = 11		Delirium Diagnosis not documented, *n* = 19				Symptoms documented, *n* = 24		Symptoms not documented, *n* = 6			
	Mean	SD	Mean	SD	*t*/*Z*	*P*‐Value	Mean	SD	Mean	SD	*t*/*Z*	*P*‐Value	Mean	SD	Mean	SD	*t*/*Z*	*P*‐Value
Age (years)	78.6	7.3	72.7	11.8	*t* = 2.3	**0.027**	82.3	5.8	76.5	7.3	*t* = 2.2	**0.034**	79.4	6.6	75.7	9.7	*t* = 1.1	0.271
MDS‐UPDRS III	55.0	16.1	48.0	15.1	*t* = 1.6	0.118	57.6	18.3	53.6	15.1	*t* = 0.6	0.537	58.3	15.9	42.2	9.2	*t* = 2.4	**0.025**
PD Duration (years)	6.5	4.3	6.4	5.1	*Z* = −0.4	0.693	6.5	5.1	6.5	3.9	*Z* = −0.3	0.747	6.3	4.4	7.0	4.0	*Z* = −0.5	0.641
LEDD (mg/day)	688.3	502.8	617.9	492.5	*t* = 1.6	0.612	790.2	609.5	629.4	437.0	*t* = 0.8	0.408	641.9	472.0	874.2	624.4	*t* = −1.0	0.32
Number of comorbidities	5.6	1.7	5.1	2.5	*Z* = −0.7	0.484	6.4	1.2	5.2	1.8	*Z* = −1.8	0.071	5.9	1.4	4.5	2.2	*Z* = −1.7	0.082
Number of medications	10.9	3.2	9.8	3.8	*Z* = −1.4	0.161	11.2	3.1	10.8	3.4	*Z* = −0.2	0.812	10.9	3.6	11.2	1.5	*Z* = −0.3	0.754
Clinical frailty scale	6.3	1.1	4.9	1.4	Z = −3.7	**<0.001**	6.5	0.8	6.2	1.2	*Z* = −0.5	0.647	6.5	0.7	5.5	1.9	*Z* = −1.5	0.139
Length of hospital stay (days)	14.7	15.1	6.2	6.0	*Z* = −2.9	**0.004**	17.6	14.9	13.0	15.4	*Z* = −1.4	0.165	16.8	16.1	6.3	5.0	*Z* = −2.0	**0.044**
MDAS total	14.7	6.2	8.0	3.6	*Z* = −4.1	**<0.001**	16.5	6.8	13.7	5.7	*Z* = −1.1	0.291	16.3	6.0	8.7	1.4	*Z* = −3.0	**0.003**
OSLA total	4.8	3.3	2.3	2.6	*Z* = −3.0	**0.003**	5.9	3.1	4.2	3.3	*Z* = −1.4	0.157	5.5	3.0	2.0	3.0	*Z* = −2.6	**0.009**
m‐RASS total	−0.6	1.8	0.2	0.752	*Z* = −1.2	0.235	0.1	2.1	−1.0	1.6	*Z* = −2.1	**0.036**	−0.8	1.9	0.0	1.1	*Z* = −0.5	0.592
Prior cognitive	1.7	1.2	1.1	0.5	*t* = 1.258	0.222	2.1	1.3	1.5	1.2	*t* = 0.775	0.451	1.8	1.2	0.9	0.7	*t* = 0.946	0.36
Impairment	*n* = 16		*n* = 8				*n* = 4		*n* = 12				*n* = 14		*n* = 2			
Gender, female	*n*	%	*n*	%	*χ* ^2^	*P*‐Value	*n*	%	*n*	%	*χ* ^2^	*P*‐Value	*n*	%	*n*	%	*χ* ^2^	*P*‐Value
	11	36.7	7	30.4	0.2	0.635	3	27.3	8	42.1	0.6	0.417	7	29.2	17	66.7	2.9	0.088

*Note*: Bold values indicates the significant differences.

Abbreviations: MDS‐UPDRS III, Movement Disorder Society Unified Parkinson's Disease Rating Scale; LEDD, Levodopa equivalent daily dose; OSLA, Observational Scale of Level of Arousal; m‐RASS, modified Richmond Agitation and Sedation Score; and MDAS, Memorial Delirium Assessment Scale.

### 3.1 Delirium documentation

The mean age of patients with ‘delirium’ documented was significantly higher than those without (mean 82.3 ± 5.8 vs. 76.5 ± 7.3 years, *p* = 0.034, Table 1). Only 11 admissions (37.9%) had a delirium diagnosis documented in their medical notes. All 11 patients with a diagnosis of delirium also had symptoms documented (100% vs. 0%, *χ* ^2^ = 11.0, and *p* = 0.001) (Table 1). All documentations of the term ”delirium” except for one was made by a doctor. Where another professional used the term delirium this was written by a physiotherapist, and this was dated 4 days prior to the term ‘delirium’ being used by a doctor. One patient was diagnosed with delirium by a doctor seen at an outpatient clinic appointment during their hospital admission. Mean time from documentation of first symptom presentation to documentation of delirium diagnosis was 1.6 ± 4.4 days. Where nursing staff or an allied health‐care professional was the first person to note a delirium symptom the mean delay in diagnosis was 4.5 ± 2.5 days, compared with 1.0 ± 0.6 days when a doctor documented the first symptom (*p* = 0.286). If the initial symptom was documented by a doctor then they were significantly more likely to have ”delirium” also documented (*n* = 9, 75% documented delirium when the first symptom by doctor vs. *n* = 2, 20% documented delirium when first symptom was allied health‐care professional *p* = 0.001).

### 3.2 Delirium subtypes

**FIGURE 2:**
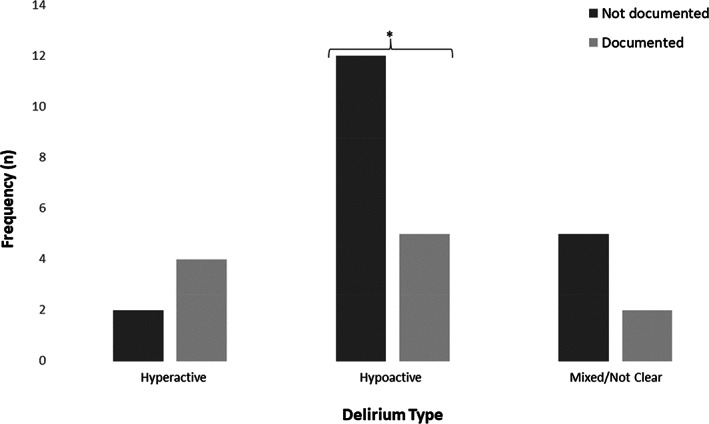
Documentation of delirium, by delirium subtypes; *p = 0.016

Participants with delirium were subtyped as hypoactive (57%, *n* = 17), hyperactive (20%, *n* = 6), mixed/unclear (23%, *n* = 7). Hypoactive delirium was significantly less likely to have been identified and formally diagnosed (63% of not documented were hypoactive vs. 37% which were hyperactive, mixed, or unclear, *p* = 0.016, Figure 2).

### 3.3 Delirium symptoms

In the medical notes delirium symptoms were documented in 24 (75%) admissions (Table 1). The first symptom was documented by doctors (59%), nursing staff (32%) or allied health‐care professionals (9%). The time from admission to the recording of the first symptom ranged from 0 days (on the day of admission) to 6 days after admission (mean 1.75 ± 1.8 days). Participants with documentation of symptoms had a significantly longer hospital admission compared to those who did not (mean 16.8 ± 16.1 vs. 6.3 ± 5 days, respectively, *p* = 0.044, Table 1). Symptom documentation was associated with significantly worse PD motor symptoms (MDS‐UPDRS III, p = 0.041) and delirium symptoms (MDAS p = 0.003, and OSLA *p* = 0.009, Table 2). Eleven admissions had a resolution of symptoms documented with total days of symptoms ranging from 1 to 15 days (mean 5.5 ± 4.6 days).

**TABLE 2:** Symptom frequency at single study assessment visit compared with documentation of symptoms in medical notes

	Single study assessment visit	Medical notes		
Confusion	n(%)	*n*(%)	*χ* ^2^	*p*‐value
Any confusion	30(100)	23(76.7)	**8.1**	**0.004**
From collateral history	22(73.3)	2(6.7)		
Reduced consciousness MDAS score ≥1	21(70)			
Disorientation MDAS score ≥1	24(80)			
Disorganized thinking MDAS score ≥1	26(86.7)			
*Hallucinations*				
Any hallucination	16(53.3)	3(10)	0.2	0.626
Complex	10(33.3)			
Illusions	4(13.3)			
Minor feeling of presence	3(10)			
Minor shadow	1(3.3)			
Simple	1(3.3)			
Auditory	7(23.3)			
*Delusions*				
Any delusion	10(33.3)	2(6.7)	1.1	0.301
Grandiose delusion	1(3.3)			
Persecutory delusion	5(16.7)			
Cotards	0(0)			
Bizarre delusion	2(6.7)			
Somatic delusion	0(0)			

*Note*: Significant results highlighted in bold.

Abbreviation: MDAS, Memorial Delirium Assessment Scale.

At the single study visit, acute confusion was the most common symptom identified (*n* = 30, 100%), followed by hallucinations (*n* = 16, 53.3%) and delusions (*n* = 10, 33.3%, Table 2). The most common symptom documented in the medical notes was also ”confusion” (*n* = 23, 76%), followed by hallucinations (*n* = 3, 10%) and delusions (*n* = 2, 6.7%, Table 2). In 23.3% of admissions, confusion was identified at the single assessment visit but not documented in the medical notes. Similarly, 46.7% of hallucination symptoms and 33.3% of delusion symptoms identified in the single research visit were not recorded in the medical notes (Table 2).

### 3.4 Discharge summaries

Of the 30 admissions with delirium, three (10%) admissions had no discharge summary completed. Where documentation of the delirium had not occurred during admission (*n* = 19), there was also no documentation of a delirium in the discharge summary. Of the 11 admissions where delirium diagnosis had been documented, only three (27%) made mention of this in the discharge summary. A plan for follow up for their confusion was documented in only two letters; however, only one of these made mention of the delirium diagnosis in the discharge letter.

## 4 DISCUSSION

Although delirium is common in PD admissions, we found that documentation of delirium is poor. Documentation of symptoms of delirium is more common but failed to lead to a formal diagnosis in over half of admissions with delirium, and there was a mean delay of 1.6 ± 4.4 between a symptom being noted and a diagnosis being made. This delay was longer when the first symptom was noted by a nurse or allied health professional; however, these results are based on small sample sizes (only 11 patients with delirium documented) and this difference did not reach significance (*p* = 0.286). Whilst the delay was not found to be significant between these two groups it was significant that when initial symptom was made by an allied health‐care professional they were less likely to go on to have a formal delirium diagnosis documented. This is suggestive that the problem does not wholly lie with the ability of staff to detect the common symptoms of delirium but also with communication of these symptoms between professionals and making a connection between these and the relevant diagnosis.

Only one of our participants had the term ”delirium” documented by someone other than a doctor. Perhaps this reflects differences in delirium training and knowledge between doctors and other health‐care professionals, with doctors using the term delirium more frequently maybe reflecting more in‐depth knowledge and training, and therefore confidence, to document this in the notes. Whilst this may be the case, it remains concerning that nursing and allied health‐care staff documentation of relevant symptoms is still failing to lead to a diagnosis being made, and again suggests that these are not being effectively communicated to the relevant medical staff such that a diagnosis can be identified and acted on.

It is possible that common delirium symptoms are being attributed to chronic PD symptoms, thus, delirium is being missed. However, in the previous study of general inpatients by Collins et al.,^24^ 72% of delirium was not documented in medical notes in a cohort of 710 medical admissions, suggesting that our findings here are not specific to Parkinson's disease but part of a wider problem with under‐detection of delirium. This is significant as Kakuma et al. identified in their study of all older adults discharged from the emergency department that those where delirium was not diagnosed had the highest mortality over the following 6 months (30.8%).^14^ PD diagnoses were not specifically looked at in this study so it remains unknown if there is any difference in outcomes for patients with PD and delirium.

Hypoactive delirium was, as expected, the more frequently missed delirium subtype. This is of concern as hypoactive delirium is associated with greater morbidity and mortality.^7^, ^25^ Along with the poor rate of diagnosis documentation, this highlights the need for improved education and awareness of delirium, subtypes, symptom profiles in PD and the importance of recognizing this and documenting it.

The documentation of delirium symptoms was associated with longer hospital admissions. Where symptoms were not documented, patients had lower PD motor symptom severity, delirium severity, and better levels of arousal. It is therefore most likely that for those admissions where delirium severity was lower, the symptoms were more likely to be missed, but also their hospital stays likely to be less complicated and thus shorter. However, in a study of postoperative outcomes from hip fracture repair by Marcantonio et al. they described a group of patients with ”subsyndromal delirium.”^6^ Whilst these patients did not meet the full Confusion Assessment Method (CAM)^26^ criteria, it was noted that if they had some symptoms of delirium then they experienced worse or similar outcomes to those with mild CAM‐defined delirium. This highlights the importance of identification of all delirium symptoms as even when not reaching diagnostic criteria (according to CAM) the outcomes can still be worse. It is also important for allowing for early interventions for preventative measures which can be a powerful and valuable way to reduce downstream consequences.^9^

Discharge letter documentation was the most notable in its lack of delirium documentation, with only a tenth of letters providing evidence of the delirium present during the hospital stay. The presence of delirium is an important piece of information for colleagues in Primary Care. An episode of delirium is associated with increased risk of future dementia but also further occurrences of delirium.^12^, ^13^ Rieck et al. highlighted that discharge summary documentation for all patients (and we would argue is still relevant to those with PD) should include not only the diagnosis of delirium but also information on effective management strategies. This can include nonpharmacologic measures such as reassurances, phrases used, music or distractions that worked well, but also any pharmacologic measures that were required and whether these were tolerated and/or effective.^9^ Improved communication and transfer of this information better allows primary care doctors and community teams to identify those patients at risk of developing dementia, but also those at risk of further delirium. Early interventions and the use of management strategies known to be helpful, and avoidance of those known to not have previously helped, for these patients, to prevent and/or minimize delirium symptoms could significantly improve their risks of morbidity and mortality.

Our study cohort was small and therefore this limits the power of our results. At the time of this study the notes we reviewed were paper files and we reviewed all paperwork including medical and nursing notes for all admissions, and the written term ”delirium” by any health‐care professional was taken to be a diagnosis. There remains a possibility that some items of paperwork were missing, however on reviewing of the patient admissions there were no noticeable discrepancies to suggest this. This form of data collection also does not account for patients where a diagnosis has been verbally recognized by the medical team and discussed but not documented. Our single research visit provided information at that time on the likely presence of delirium but the remaining further incident cases were reliant on the medical notes. Therefore, it cannot be certain whether patients had further incident delirium that would have been missed based on lack of sufficient documentation of symptoms.

In summary, our results show that delirium in PD is common but frequently missed. The most common symptom present and noted was confusion and the most commonly missed subtype of delirium was hypoactive delirium. There is lack of communication of a delirium diagnosis in the inpatient medical notes and into discharge summaries. There is a need for larger studies to accurately describe the phenomenology of delirium in PD. From this education and screening tools about delirium symptomatology and diagnosis in PD can be developed. Improving identification and documentation of delirium could reduce morbidity and mortality, whilst in hospital and following discharge. Improving the communication of the presence of delirium using hospital discharge letters would also help primary care colleagues identify patients at greater risk of dementia and further delirium.

### PEER REVIEW

The peer review history for this article is available at https://publons.com/publon/10.1111/acps.13470.

## Data Availability

The data that support the findings of this study are available from the corresponding author upon reasonable request.
